# Development of the UK Rheumatology Occupational Therapy Capabilities Framework: a national consensus initiative

**DOI:** 10.1093/rap/rkag038

**Published:** 2026-03-28

**Authors:** Yeliz Prior, William J Gregory, Simone Battista, Patricia Bisset, Sandi Derham, Dervil M Dockrell, Caroline Livesey, Sandra Lyle, Sushil Munakhya, Rachael Murphy, Gemma O’Callaghan, Katie O’Donnell, Janet Perkins, Claire Pidgeon, Helen Steer, Sally Tuson, Emma Walker, Gemma Williams

**Affiliations:** School of Health and Society, Centre for Movement and Rehabilitation, University of Salford, Salford, UK; Rehabilitation Department, Salford Royal Hospital, Northern Care Alliance NHS Foundation Trust, Salford, UK; School of Health and Society, Centre for Movement and Rehabilitation, University of Salford, Salford, UK; Rheumatology Department, Salford Royal Hospital, Northern Care Alliance NHS Foundation Trust, Salford, UK; Faculty of Health and Education, Manchester Metropolitan University, Manchester, UK; School of Health and Society, Centre for Movement and Rehabilitation, University of Salford, Salford, UK; Rheumatology Department, NHS Greater Glasgow and Clyde, Glasgow, UK; RNHRD and Brownsword Therapies Centre, Royal United Hospitals Bath NHS Foundation Trust, Bath, UK; Bone Research Group, Institute of Genetics and Cancer, University of Edinburgh, Edinburgh, UK; Occupational Therapy Service, Department of Rheumatology, Blackpool Teaching Hospitals NHS Foundation Trust, Blackpool, UK; Community, Frailty & Therapy Services, Northern Lincolnshire and Goole NHS Foundation Trust, Scunthorpe, UK; Physiotherapy Outpatient, University Hospital North Tees & Hartlepool Foundation NHS Trust, Hartlepool, UK; Rheumatology Therapy Team, Sherwood Forest Hospitals NHS Foundation Trust, Nottinghamshire, UK; Rehabilitation Department, Freeman Hospital, Newcastle Upon Tyne Hospitals NHS Foundation Trust, Newcastle upon Tyne, UK; Occupational Therapy Department, University Hospital Southampton NHS Foundation Trust, Southampton, UK; MSK Therapies Department, Manchester University NHS Foundation Trust, WTWA, Trafford General Hospital, Trafford, UK; Occupational Therapy Service, Birmingham Children’s Hospital, Birmingham, UK; MSK Outpatients Department, Sheffield Teaching Hospitals NHS Foundation Trust, Sheffield, UK; Therapies Outpatients Department, Royal Cornwall Hospitals NHS Trust, Truro, UK; MSK Outpatients Department, Sheffield Teaching Hospitals NHS Foundation Trust, Sheffield, UK; Outpatient Therapies, East Kent Hospitals University NHS Foundation Trust, Kent, UK

**Keywords:** occupational therapy, capabilities, professional standards, rheumatology rehabilitation, competencies, framework

## Abstract

**Objectives:**

To describe the development and national consensus process underpinning the Rheumatology Occupational Therapy Capabilities Framework. This work addresses the absence of a structured framework defining the knowledge, skills and professional behaviours required of the occupational therapy workforce working within rheumatology services managing rheumatic and musculoskeletal diseases and the need for a consistent national reference to support practice, workforce planning and professional development.

**Methods:**

A five-stage mixed methods process was adopted between May 2023 and September 2025, beginning with the design of a UK-wide scoping survey of rheumatology occupational therapists. Subsequent stages included expert consultation, alignment with national and international standards, stakeholder review and endorsement by professional bodies.

**Results:**

The process achieved national consensus across rheumatology occupational therapists from all four UK nations and resulted in a four-domain, five-level capability framework mapped to NHS Agenda for Change bands and allied health professions practice levels.

**Conclusion:**

This study describes a structured national consensus process used to develop and endorse a specialist capability framework for occupational therapy in rheumatology. The five-stage approach achieved formal endorsement from the Royal College of Occupational Therapists and the British Society for Rheumatology and offers a transparent, transferable model for specialist capability framework development across allied health professions.

Key messages

- A five-stage national consensus process developed a specialist rheumatology occupational therapy framework.
- Combining workforce scoping, expert consensus and stakeholder consultation strengthened methodological rigour.
- The development model offers a transferable blueprint for specialist capability frameworks.

## Introduction

Occupational therapists (OTs) play an integral role in multidisciplinary team (MDT) management of rheumatic and musculoskeletal diseases [1, 2]. Their practice focuses on supporting individuals to participate in meaningful daily activities, manage fatigue and pain and maintain employment and social roles [2–5]. Despite this essential contribution, the scope and visibility of rheumatology OT across the UK have remained variable, with limited structured guidance to support professional development and workforce planning [3, 6].

Recent policy developments, such as the National Health Service (NHS) Long Term Workforce Plan (2023) [7] and the Allied Health Professions (AHPs) Strategy for England 2022–2027 [8], have placed strong emphasis on sustainable workforce growth and capability development across AHPs. Both documents highlight the importance of capability frameworks in supporting career progression, service transformation and the expansion of advanced and consultant-level practice [7, 8]. Similarly, the Modernising Allied Health Professional Careers Programme outcome calls for maximising workforce potential by ensuring professionals operate at the top of their competence [9]. The development of the Rheumatology Occupational Therapy Capabilities Framework (ROTCF) aligns with these national ambitions by providing a structured tool to support career-long learning, retention and consistency of practice within rheumatology services.

The need for clear capability frameworks has also been underscored by workforce evidence within rheumatology itself. The British Society for Rheumatology (BSR) workforce report, A Crisis in Numbers [10], identified widespread shortages across rheumatology MDTs, including limited access to OTs. More than half of UK rheumatology services reported having no dedicated OT within their MDT, despite National Institute for Health and Care Excellence (NICE) guidance recommending access to specialist OT input with periodic review if people have difficulties with any of their everyday activities or problems with hand function [11]. This underlines both the workforce pressures facing rheumatology and the need for mechanisms that attract, retain and develop rheumatology OTs through structured professional pathways.

In this context, the BSR commissioned development of the ROTCF in partnership with academics from the University of Salford and OTs working in rheumatology across the UK. The purpose was to define the specialist occupation-centred knowledge, skills, values and behaviours required to deliver high-quality rheumatology care, aligned with the Royal College of Occupational Therapists (RCOT) professional standards and the wider NHS workforce priorities.

This article reports the structured national consensus process underpinning the ROTCF and its professional endorsement rather than reproducing the full framework document. The full framework is freely accessible via the websites of the BSR and the RCOT. Given the absence of a gold standard for capability framework development, this article emphasises methodological transparency and transferability for specialist AHPs.

## Methods

The ROTCF followed a five-stage mixed methods process between May 2023 and September 2025. While there is no apparent ‘gold standard’ or standardised approach to competency or capability framework development [12], our approach mirrored that used in the development of the Rheumatology Physiotherapy Capabilities Framework [13], which was endorsed by the BSR and the Chartered Society of Physiotherapy (CSP), to ensure methodological consistency across professions. The process was also aligned with the EULAR recommendations for the core competencies of health professionals in rheumatology [14].

### Governance and oversight

The project was jointly led by the University of Salford and the BSR, with oversight from an expert working group and a research fellow. The working group ensured that the process was transparent, inclusive and aligned with both professional standards and workforce policy priorities. The development process combined quantitative and qualitative methods, incorporating iterative consensus rounds to refine the framework through successive stages.

### Ethical approval

The scoping survey received university research ethics approval as reported in the published study [3]. Subsequent consensus and framework development activities did not require additional research ethics approval.

#### Stage 1: Scoping survey

An initial working group was convened through the BSR in May 2023. A national scoping survey of UK rheumatology OTs was undertaken to explore current roles, expertise and development needs. The survey was delivered online via Jisc and disseminated nationally through the BSR, RCOT and professional networks. Full methodological details are reported in the published scoping survey [3]. Expressions of interest were received from OTs across all four UK nations and from varied clinical settings and career stages, supporting broad representation within the expert group. Eighty-eight rheumatology OTs responded to the survey. As dissemination occurred through open professional networks and snowball sampling, the total number of practitioners who received the invitation could not be determined. As national workforce data indicate ≈87 UK rheumatology departments include provision of an OT, responses are likely to represent a substantial proportion of the specialist workforce [3]. Most respondents worked in acute hospital settings and at NHS Agenda for Change (AfC) bands 6–7, the UK national pay and grading system for NHS staff [15]. While confidence in clinical skills was high, 23% reported insufficient formal training for their role and 11% noted limited familiarity with outcome measures or patient education competencies [3]. These findings confirmed the need for a structured, capability-based framework to guide professional development and ensure consistency in practice standards [3].

#### Stage 2: Expert working group

Following the national scoping survey, 32 OTs expressed an interest in joining the expert working group, representing a range of clinical settings, career stages and areas of expertise, including both adult and paediatric rheumatology across the UK. All were invited to participate. Survey findings were descriptively analysed and synthesised by two researchers (Y.P. and S.B.) to identify key capability themes, which were presented to the group to inform initial domain development.

Three structured virtual workshops were held between September 2024 and April 2025, facilitated by the project team. Sixteen rheumatology OTs with substantive clinical roles in NHS services across the UK were able to participate consistently in the consensus and refinement stages and formed the final expert group. Draft capability domains and statements were revised iteratively across workshops and interim electronic circulation rounds. Following each round, proposed amendments were collated and recirculated. Revisions were incorporated and consensus was considered achieved when no unresolved objections remained.

The working group also mapped stakeholder groups and areas of application for the framework (Table 1) to ensure relevance across clinical, educational, managerial and policy contexts.

**Table 1:** Stakeholders in the capabilities framework and areas of application (occupational therapy workforce, managers, clinical commissioners, educational commissioners, national regulators, and public and patients).

Occupational therapy workforce (including support workers)	Managers	Clinical commissioners	Educational commissioners	National regulators	Public and patients
Professional progression is measured against a specific national framework	Provides information to support business cases to improve rheumatology and/or occupational therapy provision	Provides benchmarking to help identify quality markers for service definition and review	Provides a framework to identify knowledge and skill gaps for the occupational therapy workforce	Provides a framework for leadership and accountability	Enable the public to understand the evolving role of the occupational therapy support worker and OTs involved in the care of individuals with rheumatologic conditions
Provides a framework for structured and informal reflective practice and continuous professional development	Provides a reference document to support workforce development and clinical supervision	Increased understanding of occupational therapy capabilities	Provides clarity of expected skills required to increase employability of postgraduates	Provides a framework that is linked to other national and international rheumatology frameworks and standards	Improves public understanding of the OT and occupational therapy support workers roles within the context of the rheumatology MDT
Creates a common language to improve communication across the occupational therapy workforce	Helps with clarification on required levels of practice in rheumatology occupational therapy	Provides a national reference point for rheumatology services and highlights transferable skills that can be applied across wider health and social care settings	Provides a framework to inform the selection and review of educational placements	Workforce development can be reviewed and benchmarked	Supports a dialogue between the public and the occupational therapy workforce in rheumatology
Establishes a framework for constructive feedback to inform clinical supervision and appraisal review at specified intervals	Provides a greater understanding of generic skills to maximise efficiency of care	Provides a framework to help prioritise investment in occupational therapy provision	Highlights the importance of measurement, audit and research in the development of occupational therapy in this field	Targeted engagement with speciality experts to improve service regulation	Patients and public can support workforce change with a more informed perspective leading to co-production

Adapted from https://ics.ac.uk/guidance/ahp-critical-care-capability-framework.html (accessed November 2025).

#### Stage 3: Alignment with national and international standards

The draft framework was cross-referenced against key national and international professional frameworks to ensure consistency with established standards for workforce development, education and MDT practice in rheumatology and occupational therapy. These included BSR professional and quality standards [16] that define expectations for MDT rheumatology practice and workforce capability; Getting It Right First Time (GIRFT) Rheumatology National Report [17] produced by NHS England with input and support from the BSR, which provides system-level recommendations for improving service delivery and workforce planning in rheumatology; EULAR recommendations for the generic core competencies of health professionals in rheumatology [14] and RCOT professional frameworks including the Career Development Framework: Guiding Principles for Occupational Therapy [18] and the Professional Standards for Occupational Therapy Practice, Conduct and Ethics [19].

Cross-referencing against these key publications provided clarity on professional scope, indicative AfC banding and expectations, and ensured alignment with national workforce policy, professional standards and international competency expectations, providing assurance that the capabilities reflect contemporary, evidence-informed and occupation-centred rheumatology practice.

The framework adopted five levels of practice, consistent with existing AHP frameworks (Table 2), providing clarity on scope, indicative AfC bands [15] and expectations at each level of capability.

**Table 2:** Levels of practice recognised across AHP frameworks, with indicative NHS AfC bands and typical scope of practice.

Levels of practice	Indicative AfC band(s)	Typical scope
**Support workforce (assistant practitioner/senior OT support worker)**	Band 3–4	Occupational therapy support workers or assistant practitioners exercising autonomy under supervision within defined parameters of practice
**Foundation practitioner**	Band 5	Newly registered OTs, working at the foundation level. This may include those entering the profession for the first time, returners to practice, international recruits and OTs transitioning from another area of practice
**Enhanced/specialist**	Band 6–7	Complex cases, consolidated skills and knowledge, splinting and vocational interventions, supervision/teaching, service improvement
**Advanced practitioner**	Band 8a	Advanced clinical leadership, pathway development, research/audit leadership, consultancy
**Consultant practitioner**	Band 8b	Strategic/system leadership, national guidance, service innovation and workforce planning

#### Stage 4: Stakeholder consultation

The draft framework was circulated for national consultation with practising rheumatology OTs and wider professional and patient stakeholders. This included OTs who had expressed interest in joining the expert working group during stage 1, as well as members of the RCOT and BSR clinical networks. Stakeholder organisations included Arthritis UK, the National Rheumatoid Arthritis Society, the National Axial Spondyloarthritis Society, the British Psoriatic Arthritis Consortium and the British Society for Spondyloarthritis.

Stakeholders were invited to comment on clarity, completeness, clinical relevance, band progression and alignment with professional standards. Input from patient organisations helped ensure that the framework language reflected principles of person-centred care, inclusivity and equity. Feedback was submitted in writing by e-mail and collated into a structured summary document. Comments were grouped thematically (e.g. terminology, scope, clinical content, leadership and governance). Proposed amendments were incorporated where consistent with consensus principles and recirculated to the expert group for a further review prior to finalisation.

#### Stage 5: Endorsement and dissemination

The final version of the framework was submitted to the BSR Education Committee and to the RCOT for formal review. Following an iterative review process, the framework received endorsement from both the BSR and the RCOT, confirming its alignment with national professional standards and workforce development priorities.

## Results

The five-stage development process resulted in a nationally agreed upon capability framework defining the occupation-centred contribution of occupational therapy within rheumatology services. Across three structured virtual workshops and three draft iterations, domains and capability statements were progressively refined through facilitated discussion and interim electronic circulation. Sixteen rheumatology OTs participated consistently in the consensus and refinement stages.

Through iterative consensus, the expert group identified four interdependent domains as the core pillars of rheumatology occupational therapy practice: clinical knowledge and understanding of rheumatologic conditions; assessment, intervention planning and evaluation; governance, quality and evidence-based practice; and leadership, education and workforce development. The inclusion of governance and leadership as distinct domains reflected agreement that contemporary rheumatology occupational therapy practice extends beyond direct clinical care to encompass service development, workforce capability and system-level influence.

Each domain is articulated across five levels of practice aligned to NHS AfC bands [15] and AHP practice levels [20]. No domains were removed following wider stakeholder consultation. However, external feedback led to refinement of occupation-centred language, clarification of band progression and expansion of clinical detail including vocational rehabilitation, transition care, medication impact and audit responsibilities. Minor amendments were made prior to formal endorsement.

The consensus process underscored the need for flexibility and aspiration within the framework. Given the variation in rheumatology service models across the UK, the expert group agreed that the framework must support local adaptation while maintaining nationally agreed upon standards. It defines the levels of practice that OTs should work towards, recognising that service configuration, workforce composition and geographical context may influence implementation. This dual focus ensures that the framework serves as both a practical reference and a structured pathway for professional progression.

The final framework received formal endorsement from the BSR and the RCOT. The full framework document, including detailed capability statements, is accessible via the websites of the BSR and the RCOT.

## Discussion

This article describes a structured, five-stage approach to developing a profession-specific capabilities framework within a subspecialist area of practice. In the absence of a universally accepted methodology for capability framework development, transparent reporting of the process is essential. The approach combined national workforce scoping data, iterative expert consensus, alignment with existing standards and multistakeholder consultation, culminating in formal endorsement from professional organisations. This layered design strengthened both professional legitimacy and ecological validity.

A key strength of the process was the integration of bottom-up and top-down influences. The initial scoping survey ensured that the framework reflected contemporary practice realities across UK rheumatology services, while subsequent alignment with national and international standards ensured coherence with existing professional guidance. The iterative workshop structure allowed refinement of language, clarification of band progression and strengthening of occupation-centred identity in response to stakeholder feedback. The absence of domain removal following consultation suggests conceptual stability, while documented refinements demonstrate responsiveness.

The consensus process also highlighted the importance of balancing standardisation with flexibility. Variation in workforce configuration and service models across the UK required a framework that defined aspirational levels of practice while allowing local adaptation. This dual focus reflects broader challenges in specialist workforce development across allied health professions.

The development process shares similarities with other rheumatology capability initiatives, including the Rheumatology Physiotherapy Capabilities Framework [13], which also adopted structured expert consensus and alignment with national workforce structures. However, the OT context introduced distinct service-level considerations. In many settings, rheumatology OT provision is delivered by small teams or single clinicians, influencing how capability levels are operationalised locally. As such, implementation may require tailored approaches within different governance and supervision systems. Compared with the generic EULAR core competencies for health professionals in rheumatology, which articulate shared interprofessional standards, this framework translates broad principles into profession-specific, banded capability statements aligned to UK workforce structures. A planned 3-year review will evaluate implementation, workforce impact and service outcomes, enabling iterative refinement in response to real-world application.

This study has limitations. Participation in the expert group was voluntary and therefore subject to self-selection bias. Although representation spanned all four UK nations and multiple service contexts, consensus methods cannot eliminate professional perspective bias. The process did not employ formal Delphi voting or quantitative agreement thresholds; instead, consensus was defined pragmatically through iterative refinement and absence of unresolved objections. While appropriate for professional framework development, this approach prioritises collective agreement over statistical measurement.

The five-stage development model described may offer a transferable template for other specialist disciplines seeking to define capability frameworks grounded in practice reality and aligned with national standards. Future evaluation should examine implementation uptake, workforce impact and service-level outcomes to determine the longer-term effectiveness of the framework.

## Conclusion

This article describes a structured national consensus process used to develop and endorse a specialist capability framework for occupational therapy in rheumatology. The five-stage, evidence-informed approach achieved formal endorsement from the BSR and the RCOT and established a nationally agreed upon standard for workforce development and professional progression. This transparent methodology may serve as a transferable model for specialist capability framework design across allied health professions. A planned 3-year review will evaluate implementation and workforce impact to support ongoing refinement.

## Data Availability

The anonymised data underlying this article will be shared upon reasonable request to the corresponding author.
